# Process Improvement of In-Hospital Critical and Life-Threatening Patient Resuscitation: A Quality Improvement Project in a China Otolaryngology Hospital

**DOI:** 10.3390/healthcare14101306

**Published:** 2026-05-12

**Authors:** Xiaoyu Weng, Chao Fang, Wenyan Li, Shuxin Xi, Hongmeng Yu, Yuejun Wu

**Affiliations:** 1 Eye & ENT Hospital, Fudan University, Shanghai 200031, China; xiaoyu.weng@fdeent.org (X.W.); chao.fang@fdeent.org (C.F.); wenyan_li@fudan.edu.cn (W.L.); shuxinxi71@126.com (S.X.); 2 ENT Institute and Otorhinolaryngology Department of Eye & ENT Hospital, Shanghai 200031, China; 3 National ENT Healthcare Quality Management Center, Shanghai 200031, China

**Keywords:** emergency, resuscitation, quality improvement

## Abstract

Background: To identify factors contributing to the low success rate of resuscitation, we optimized related links in resuscitation management and constructed a five-minute green transfer resuscitation model. Methods: A quasi-experimental pre–post quality improvement study was conducted on patients with critical and severe conditions admitted in the Department of Otorhinolaryngology at a China otolaryngology hospital. The pre-intervention group of patients were treated using the conventional resuscitation process, while the post-intervention group was treated using the “5 min green transfer” resuscitation process under the guidance of the quality improvement (QI) team. Results: The resuscitation mainly occurred in the first and second quarters, between 20:00 in the evening and 07:59 the following morning. In the pre-intervention group, the most common direct cause of initiating resuscitation was bleeding, primarily due to epistaxis, while the primary direct cause for initiating resuscitation was abnormal vital signs in the post-intervention group. The resuscitation success rate was 82.93% (34/41) in the pre-intervention group and 93.48% (43/46) in the post-intervention group. However, there was no statistically significant difference in resuscitation success rate (*p* = 0.14) and complication incidence (*p* = 0.71) between the two groups. In the pre-intervention group, six patients (14.63%) were transferred within 5 min, whereas 100% of patients (46 cases) in the post-intervention group achieved 5 min transfer, with a statistically significant difference observed between the two groups (*p* = 0.03). Conclusions: The intervention significantly improved the 5 min transfer efficiency, which was conducive to ensuring timely medical intervention for patients and safeguarding their clinical safety.

## 1. Introduction

The overall incidence of perioperative complications in otolaryngology surgery ranges from 5% to 20%, with significant variation attributed to factors such as the type of surgery and the patient’s underlying conditions [1,2]. Among these complications, hemorrhage is common, severe and potentially fatal in head and neck surgery, which can lead to airway obstruction, increase infection rates and even cause hemorrhagic shock or death [3]. Recent research has suggested that the mortality rate associated with massive bleeding due to carotid artery rupture following nasopharyngeal cancer radiotherapy can reach 60% [4]. Some studies have shown that the risk of postoperative bleeding after tonsillectomy varies between 2% and 21.4% [5,6]. Moreover, evidence has shown that patients with head and neck tumors undergoing free flap reconstruction are at a particularly high risk for postoperative hemorrhage [7].

Given that it is impossible to completely prevent postoperative complications, enhancing the management of these complications and improving the emergency resuscitation capabilities for seriously ill patients are of paramount importance. Over the long term, the emergency resuscitation system comprising pre-hospital care, hospital emergency care and intensive care has become relatively well-established. However, the identification, emergency management and resuscitation of patients with critical and life-threatening conditions within the hospital still remain significant challenges [8].

Conventionally, the management of in-hospital emergency resuscitation mainly relies on experience-based clinical decision-making, passive response to acute events, and fragmented departmental collaboration—strategies that prioritize immediate symptom relief over systematic risk prevention and process optimization. These conventional approaches, however, are being increasingly deemed insufficient in the current clinical context. Specifically, during the process of emergency transfer, inadequate coordination and communication between departments, as well as information gaps in the process, can seriously impact the effectiveness of patient resuscitation and prognosis [9], which further highlights the limitations of conventional unstructured strategies.

Against this backdrop, it is imperative to develop optimized and structured quality improvement (QI) strategies featuring standardized operational protocols, systematic risk assessment mechanisms, and cross-departmental collaborative frameworks. This study employed a quasi-experimental pre–post design in the otolaryngology department of a tertiary specialized hospital in China. Driven by an institutional policy prioritizing the standardization and efficiency of clinical resuscitation, the department shifted from traditional resuscitation methods to a quality improvement (QI)-guided approach in 2024. This study aimed to identify factors associated with low resuscitation success rates, optimize key links in resuscitation management, and establish a five-minute green transfer resuscitation model under the QI team, thereby providing a valuable reference for postoperative resuscitation in other otolaryngology departments.

## 2. Materials and Methods

### 2.1. Setting and Participants

This study was conducted in the Department of Otorhinolaryngology at a China otolaryngology hospital. A total of 87 patients with critical and severe conditions were included in this study, divided into two groups according to the resuscitation process received. Specifically, a retrospective analysis was conducted on 41 patients with critical and severe conditions admitted between July 2022 and December 2023, who were treated using the conventional resuscitation process and were designated as the pre-intervention group. Another group of 46 patients with critical and severe conditions admitted between January 2024 and July 2025, who were treated using the “5 min green transfer” resuscitation process, were designated as the post-intervention group. Ethical constraints prevent the random allocation of critically ill otolaryngology emergency patients to distinct resuscitation protocols, as such practices would compromise patient safety and contravene ethical principles. Accordingly, a before–after observational design represents the most methodologically appropriate approach for this study. This design assesses intervention efficacy by comparing pre- and post-intervention clinical outcomes while ensuring feasibility and safety.

### 2.2. Inclusion and Exclusion Criteria

Inclusion criteria: Patients who experienced airway obstruction, bleeding, cardiac or respiratory arrest, abnormal vital signs, or other life-threatening or severe conditions during hospitalization, And patients without significant functional impairments in their medical history. Exclusion criteria: Patients with cognitive impairment or a history of mental illness, and patients with incomplete clinical data.

### 2.3. Study Design

The Hospital Committee for Medical Quality and Safety Management regularly meets to review the causes of in-hospital patient mortality and to identify preventable areas for improvement. A prominent concern was the low resuscitation success rate or late timing of initiating resuscitation among seriously ill patients. In response to these concerns, a quality improvement (QI) team that included otolaryngologists, QI coordinators and a hospital administrator was assembled. Adhering to the Model for Improvement Guidelines, the team spearheaded this initiative as a QI study [10].

### 2.4. Cause-and-Effect Analysis and Driver Diagram

**Figure 1:**
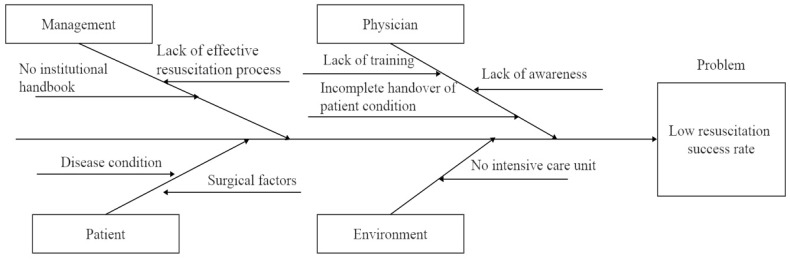
Cause-and-effect diagram.

In this study, a cause-and-effect analysis and driver diagram—a visual framework used to map causal relationships and prioritize key factors influencing process performance— was employed to identify the potential factors contributing to the low success rate of in-hospital emergency resuscitation (Figure 1). The root cause analysis was conducted from four broad categories: clinical physicians, management mechanisms, patient conditions and environmental facilities. Among these categories, the QI team identified causes as follows:

(1) Physician: Lack of emergency awareness, insufficient skill training, incomplete handover of patient conditions.
(2) Management: Absence of effective emergency protocols, lack of relevant institutional regulations.
(3) Patient: Severity of the condition, surgical factors.
(4) Environmental: Lack of critical care monitoring conditions.

### 2.5. Intervention

#### 2.5.1. Revision of In-Hospital Emergency Standard Procedures

**Figure 2:**
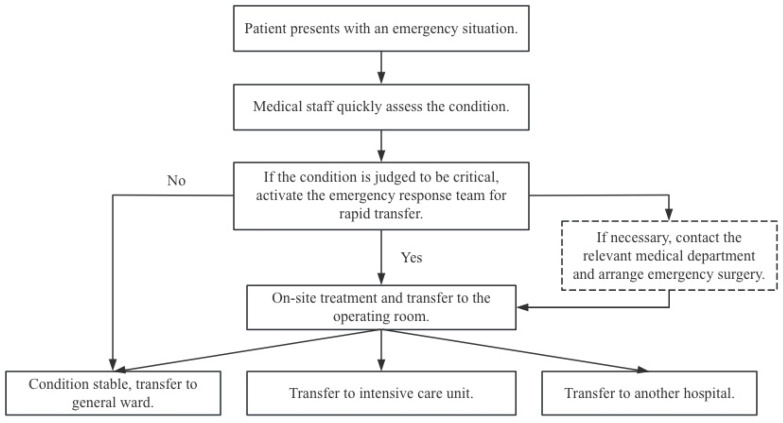
Standardized procedures for resuscitation.

Led by the QI team, the revision of the Hospital Emergency Management Manual and the Manual for the Prevention and Treatment of Major Bleeding in Otolaryngology was initiated. These revisions focused on standardizing the processes for monitoring, identifying, and activating the emergency system, on-site management and efficient transfer of patients with postoperative emergencies in otolaryngology. The team collaboration across these critical stages was meticulously reviewed to establish standardized procedures (Figure 2).

As shown in Figure 2, the workflow began when a patient presented with an emergency, followed by prompt clinical assessment by medical staff. Non-critical cases followed routine care pathways, while critical cases triggered the activation of a 5 min green channel to mobilize the emergency response team for rapid intervention. If urgent surgery was required, relevant departments were contacted to arrange emergency surgery, concurrent with on-site treatment and transfer to the operating room. Post-intervention, patients were triaged based on their clinical status: stable patients were transferred to the general ward, those requiring close monitoring were admitted to the intensive care unit, and those needing specialized care were transferred to another hospital. This standardized emergency management framework was integrated into a unified system and incorporated into clinical training and assessment protocols.

#### 2.5.2. Formation of the Rapid Response Team

A Medical Emergency Team (MET) was established in each ward, consisting of on-call clinical physicians from various specialties, anesthesiologists, and nurses. All members were required to receive Basic Life Support (BLS) training and pass assessments at an American Heart Association (AHA)-accredited training center. In addition, clinical physicians were required to demonstrate proficiency in endotracheal intubation—a skill defined as the ability to successfully insert an endotracheal tube into the trachea within a specified time frame (≤3 min), even in difficult airway scenarios, to ensure effective ventilation and oxygenation.

#### 2.5.3. Training and Drills

The QI coordinators regularly organized clinical training, with additional training and assessments in emergency scenario simulation and team-based collaboration. Among the post-intervention group, in-hospital emergency scenario simulation drills were conducted monthly, with different wards and scenarios. These drills covered all aspects, including emergency identification, initiation of emergency response, on-site management and transportation of patients in critical condition. Time points for each phase were monitored and recorded to identify weak links for continuous improvement.

#### 2.5.4. Early Warning Mechanism

The QI team leveraged information technology to upgrade the hospital information system and laboratory system, thereby enhancing the management of critical value information. Once a critical value was detected, the system automatically popped up a window to remind the laboratory staff to recheck the result. Simultaneously, it sent an alert box to the physician workstation, a text message to the relevant clinical physicians and a phone notification to the nursing station. This multi-channel and multi-level notification system for critical values ensured that clinicians receive critical value information in a timely manner.

### 2.6. Evaluation Indicators and Data Collection

Resuscitation initiation causes were classified by International Classification of Diseases, 10th Revision (ICD-10). Collected data included demographic variables (gender, age), clinical timestamps (call time, season), disease-related parameters (disease type, direct resuscitation cause), complications, and clinical outcomes. Key outcomes were explicitly defined: ① Resuscitation success: Restoration of spontaneous circulation (ROSC) ≥ 20 min post-resuscitation with stable vital signs. ② Death: Irreversible cessation of all vital functions (heartbeat, breathing, cerebral function) during/after resuscitation, confirmed by two attending physicians. ③ Complications: Adverse events during/within 72 h post-resuscitation diagnosed per clinical guidelines. ④ Transfer within 5 min: Transfer to the operating room for emergency management within 5 min of transfer order issuance. Data were collected independently by two trained researchers via EMR review, verified, and cross-checked (discrepancies resolved with supervisor). All data were anonymized with unique serial numbers; records were securely stored with restricted access, used only for this study, and managed per hospital regulations for confidentiality.

### 2.7. Statistical Analysis

A database for resuscitation cases was established using Miscrosoft Excel 2016 software, and statistical descriptions and analyses were performed using SPSS 25.0 software. Count data were presented as frequencies and proportions. Statistical analyses were conducted using statistical tables and graphs. The direct causes of resuscitation initiation were analyzed using a Pareto chart. Comparisons between frequency groups were performed using chi-square analysis, with *p* < 0.05 indicating statistical significance.

## 3. Results

### 3.1. Basic Characteristics of Patients Who Underwent Resuscitation

Among the patients who initiated resuscitation (Table 1), there were 65 males (74.71%) and 22 females (25.29%), with the majority being elderly individuals over 60 years old (57.47%). In the pre-intervention group, there were 25 males (60.98%) and 16 females (39.02%), with 22 elderly individuals over 60 years old (53.66%). In the post-intervention group, there were 40 males (86.96%) and 6 females (13.04%), with 28 elderly individuals over 60 years old (60.87%).

**Figure 3:**
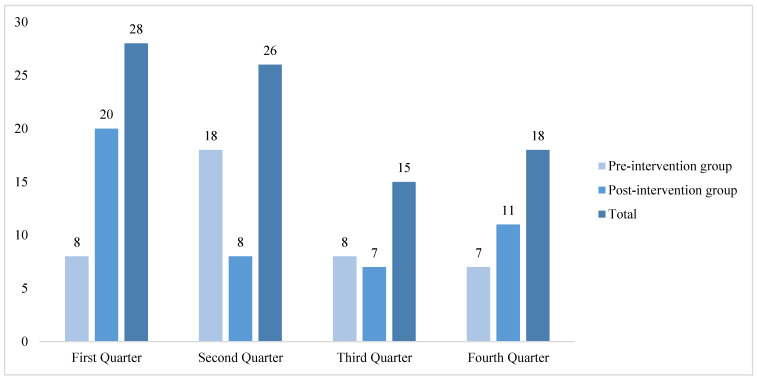
Distribution of resuscitation cases by quarter.

**Figure 4:**
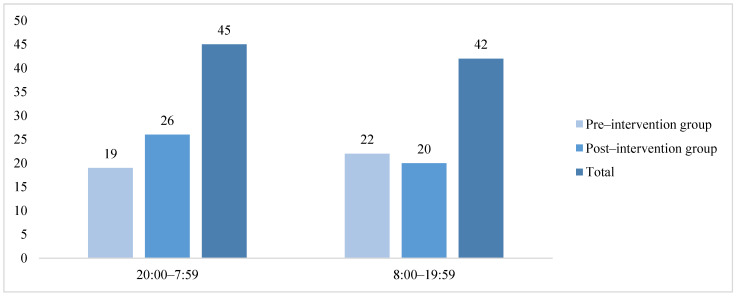
Distribution of resuscitation cases by occurrence time.

In terms of seasonal and temporal distribution (Figure 3 and Figure 4), the majority of resuscitation cases among inpatients occurred in the first quarter (28 cases) and the second quarter (26 cases). The number of resuscitation cases initiated between 20:00 and 07:59 of the following morning (45 cases) was higher than that between 08:00 and 19:59 (42 cases).

### 3.2. Comparison of Direct Causes for Initiating Resuscitation

**Figure 5:**
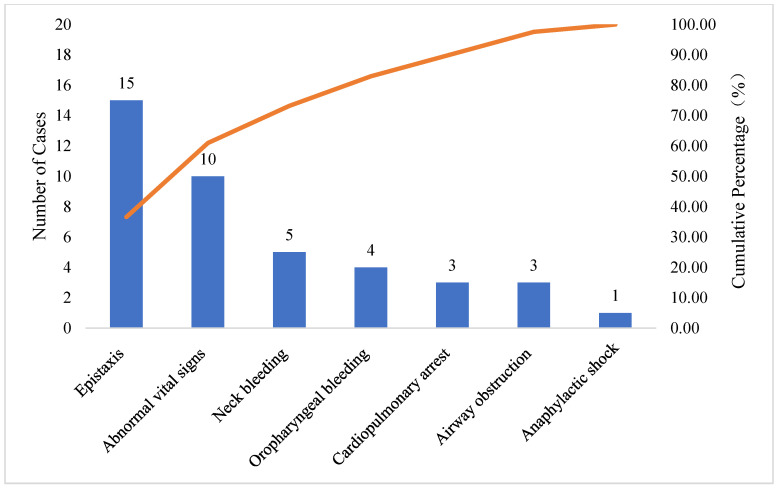
Pre-intervention group pareto chart.

**Figure 6:**
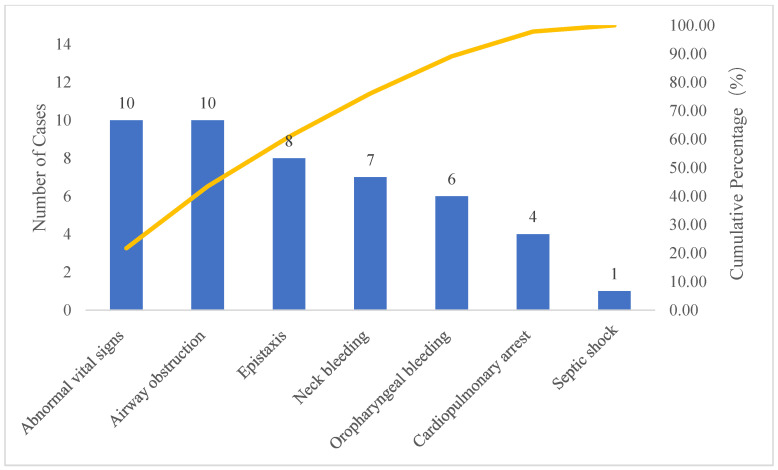
Post-intervention group pareto chart.

In the pre-intervention group (*n* = 41), epistaxis (15 cases, 37.5%) and abnormal vital signs (10 cases, 25.0%) were the most common causes, together accounting for 62.5% of cases. Neck bleeding (5 cases), oropharyngeal bleeding (4 cases), cardiopulmonary arrest (3 cases), airway obstruction (3 cases), and anaphylactic shock (1 case) were less frequent (Table 2). In the post-intervention group (*n* = 46), abnormal vital signs (10 cases, 21.7%) and airway obstruction (10 cases, 21.7%) were the leading triggers, together accounting for 43.4% of cases (Table 3). Epistaxis (8 cases), neck bleeding (7 cases), oropharyngeal bleeding (6 cases), cardiopulmonary arrest (4 cases), and septic shock (1 case) were less frequent. Direct causes for initiating resuscitation in both groups were ranked by frequency using pareto charts (Figure 5 and Figure 6).

### 3.3. Evaluation of the Effects Between Pre-Intervention and Post-Intervention Group

In the pre-intervention group, 34 cases (82.93%) were successfully resuscitated, 4 cases (9.76%) died and 3 cases (7.32%) chose to be discharged without further resuscitation. In the post-intervention group, 43 cases (93.48%) were successfully resuscitated, 3 cases (6.52%) died and no patients chose to be discharged without further resuscitation. There was no statistically significant difference in the outcomes of resuscitation between the two groups (*p* = 0.14). In terms of post-resuscitation complications, 30 cases (73.17%) in the pre-intervention group had no complications, while 11 cases (26.83%) had complications. In the post-intervention group, 32 cases (69.57%) had no complications, and 14 cases (30.43%) had complications. There was no statistically significant difference in the occurrence of complications between the two groups (*p* = 0.71). In the pre-intervention group, 6 patients (14.63%) were transferred within 5 min, whereas 100% of patients (46 cases) in the post-intervention group achieved 5 min transfer, with a statistically significant difference observed between the two groups (*p* = 0.03) (Table 4).

## 4. Discussion

### 4.1. Analysis of the Initiation of Resuscitation in Patients

In this study, most hospitalized patients requiring in-hospital resuscitation were elderly males aged 60 years and above, consistent with Shappell’s findings. Driven by societal population aging, the average age of hospitalized patients has increased globally [11]. Regarding seasonal and temporal distribution, otolaryngology-related in-hospital resuscitation events were concentrated in the first and second quarters, accounting for 62.07% of total cases, which accords with Churpek et al.’s results. Frequent temperature fluctuations in these two quarters coincide with the peak prevalence of otolaryngological diseases [12]. Moreover, 51.72% of resuscitation incidents occurred during nighttime (20:00–07:59). Existing studies have confirmed that nighttime is a high-incidence period for cardiovascular diseases [13], and medical staff fatigue at night increases the risk of delayed resuscitation for critically ill patients [14]. These results suggested that hospitals need to enhance monitoring from night to early morning and promptly initiate effective resuscitation.

### 4.2. Analysis of the Causes for Initiating Resuscitation

The identification and prevention of in-hospital critical illnesses were far more important than treatment [15]. Thus, understanding the characteristics of the causes of in-hospital critical illness resuscitation was of great significance for ultimately improving the success rate of emergency treatment. In this study, pareto charts visually highlighted the dominant factors, offering clear evidence to show direct causes for initiating resuscitation. In the pre-intervention group, epistaxis was identified as the primary targets for intervention, suggesting that medical institutions should enhance the prevention of postoperative bleeding [16,17,18]. In the post-intervention group, the direct cause for initiating resuscitation was abnormal vital signs, indicating the need to strengthen the monitoring of postoperative vital signs [19,20]. Additionally, there should be increased education for patients and their families to emphasize the importance of monitoring vital signs after surgery and to initiate resuscitation promptly.

### 4.3. Effect Evaluation of Initiating Resuscitation

Under the guidance of the QI team, a comprehensive evaluation of the pre-intervention group healthcare workers was conducted, covering emergency resuscitation protocols and patient transfer processes. A cause-and-effect analysis and driver diagram was employed for root cause analysis, identifying issues in healthcare workers, processes and systems [21]. Targeted improvement strategies were then developed and implemented. In the post-intervention group, training and drills for healthcare workers were enhanced, focusing on resuscitation and transfer timeliness, effectively shortening transfer duration and improving treatment success rates.

In the pre-intervention group, healthcare workers had relatively low awareness of preventing postoperative bleeding and made vague judgments when issuing transfer orders during resuscitation, which to some extent prolonged the transfer time for patients. In contrast, for the post-intervention group, to optimize patient transfer and treatment efficiency, the responsibilities of the resuscitation team were further clarified, and transfer routes in various wards were simplified to save resuscitation time. Notably, the intervention significantly enhanced 5 min transfer efficiency, which facilitated timely medical intervention for patients and safeguarded their clinical safety.

Consistent with our findings, the existing literature has confirmed that QI interventions optimized resuscitation workflows and enhanced clinical outcomes, supported by Leong et al., who reported that QI-driven training and process optimization shortened resuscitation duration and improved survival by mitigating human errors and systemic inefficiencies [22]. Notably, our study extended prior research [23] by establishing a five-minute green transfer resuscitation model tailored to otolaryngology postoperative resuscitation—where rapid transfer and airway management were critical.

## 5. Conclusions

This study demonstrated that the continuous quality improvement mechanism developed and implemented by the quality improvement team significantly enhanced the efficiency of the 5 min transfer process, enabling timely clinical intervention and mitigating potential patient safety risks. However, several limitations remain. First, short-term or long-term patient mortality outcomes were not examined, precluding accurate evaluation of the mechanism’s impact on treatment efficacy and patient survival. Second, notable baseline imbalance existed between the two groups, particularly in gender distribution, which may introduce confounding effects and weaken intergroup comparability. Additional methodological limitations include temporal variations in clinical practice and unmeasured confounding variables. To enhance the clinical relevance of the findings, future research should include mortality and long-term outcomes as key indicators, adopt robust grouping protocols to ensure balanced baseline characteristics, and utilize multicenter prospective designs with larger sample sizes. Such improvements will enhance the generalizability of the results for emergency resuscitation practices in otolaryngology.

## Figures and Tables

**Table 1:** Baseline characteristics (*n*/%).

	Total (*n* = 87)	Pre-Intervention Group (*n* = 41)	Post-Intervention Group (*n* = 46)	*χ* ^2^	*p* Value
Sex				7.75	<0.01
Male	65 (74.71)	25 (60.98)	40 (86.96)		
Female	22 (25.29)	16 (39.02)	6 (13.04)		
Age				0.74	0.86
<60	37 (42.53)	19 (46.34)	18 (39.13)		
60~69	30 (34.48)	14 (34.15)	16 (34.78)		
70~79	17 (19.54)	7 (17.07)	10 (21.74)		
≥80	3 (3.45)	1 (2.44)	2 (4.35)		
Quarter				9.69	0.02
First Quarter	28 (32.18)	8 (19.51)	20 (43.48)		
Second Quarter	26 (29.89)	18 (43.90)	8 (17.39)		
Third Quarter	15 (17.24)	8 (19.51)	7 (15.22)		
Fourth Quarter	18 (20.69)	7 (17.07)	11 (23.91)		
Occurrence Time				0.90	0.34
20:00–7:59	45 (51.72)	19 (46.34)	26 (56.52)		
8:00–19:59	42 (48.28)	22 (53.66)	20 (43.48)		

**Table 2:** Pre-intervention group direct cause analysis for emergency resuscitation initiation.

Cause Classification	Number of Cases	Percentage (%)	Cumulative Percentage (%)
Epistaxis	15	36.59	36.59
Abnormal vital signs	10	24.39	60.98
Neck bleeding	5	12.20	73.17
Oropharyngeal bleeding	4	9.76	82.93
Cardiopulmonary arrest	3	7.32	90.24
Airway obstruction	3	7.32	97.56
Anaphylactic shock	1	2.44	100.00

**Table 3:** Post-intervention group direct cause analysis for emergency resuscitation initiation.

Cause Classification	Number of Cases	Percentage (%)	Cumulative Percentage (%)
Abnormal vital signs	10	21.74	21.74
Airway obstruction	10	21.74	43.48
Epistaxis	8	17.39	60.87
Neck bleeding	7	15.22	76.09
Oropharyngeal bleeding	6	13.04	89.13
Cardiopulmonary arrest	4	8.70	97.83
Septic shock	1	2.17	100.00

**Table 4:** Patient outcome measures with improvement on evaluation criteria.

	Pre-Intervention Group (*n* = 41)	Post-Intervention Group (*n* = 46)	*p* Value
Resuscitation outcome			0.14
Death	4 (9.76%)	3 (6.52%)	
Success	34 (82.93%)	43 (93.48%)	
* Self-discharge	3 (7.32%)	0 (0.00%)	
Complications			0.71
No	30 (73.17%)	32 (69.57%)	
Yes	11 (26.83%)	14 (30.43%)	
Transfer within 5 min			0.03
No	35 (85.37%)	0 (0.00%)	
Yes	6 (14.63%)	46 (100.00%)	

* Self-discharge means against medical advice following the family’s decision to abandon resuscitation.

## Data Availability

The datasets generated during this study are available from the corresponding author on reasonable request.

## Abbreviations

The following abbreviations are used in this manuscript:

QI	Quality improvement
MET	Medical emergency team
BLS	Basic life support
AHA	American Heart Association
ICD-10	International Classification of Diseases, 10th revision
